# Advances in Immunotherapy for Endometrial Cancer: Insights into MMR Status and Tumor Microenvironment

**DOI:** 10.3390/cancers16233918

**Published:** 2024-11-22

**Authors:** Manel Albertí-Valls, Sara Olave, Anna Olomí, Anna Macià, Núria Eritja

**Affiliations:** 1 Oncologic Pathology Group, Biomedical Research Institute of Lleida (IRBLleida), University of Lleida (UdL), Av. Rovira Roure 80, 25198 Lleida, Spain; 2 Centro de Investigación Biomédica en Red de Cáncer (CIBERONC), 28029 Madrid, Spain; 3 Developmental and Oncogenic Signaling, Biomedical Research Institute of Lleida (IRBLleida), University of Lleida (UdL), Av. Rovira Roure 80, 25198 Lleida, Spain

**Keywords:** endometrial cancer, immunotherapy, mismatch repair, tumor mutational burden, tumor microenvironment, immune checkpoint blockade, cell-based therapies

## Abstract

Endometrial cancer is one of the most common gynecological malignancies, and while early-stage cases are highly treatable, recurrent or advanced EC remains challenging to manage. Immunotherapy, particularly immune checkpoint inhibitors, has revolutionized treatment approaches in oncology, and its application in EC has shown promising results. Key to immunotherapy efficacy in EC is the tumor’s mismatch repair status, with MMR-deficient tumors demonstrating a higher tumor mutational burden and increased PD-L1 expression, making them more susceptible to immune checkpoint inhibitors (ICIs) such as pembrolizumab, durvalumab, and dostarlimab. However, not all mismatch repair-deficient (MMRd) tumors respond to ICIs, particularly those with a “cold” tumor microenvironment (TME) characterized by poor immune infiltration. In contrast, some MMR-proficient tumors with a “hot” TME respond well to ICIs, underscoring the complex interplay between MMR status, tumor mutational burden (TMB), and TME. To overcome resistance in cold tumors, novel therapies, including Chimeric Antigen Receptor (CAR) T cells and tumor-infiltrating lymphocytes are being explored, offering targeted immune-based strategies to enhance treatment efficacy. This review discusses the current understanding of immunotherapy in EC, emphasizing the prognostic and therapeutic implications of MMR status, TME composition, and emerging cell-based therapies.

## Simple Summary

Endometrial cancer (EC), while generally curable in early stages, poses significant challenges when it recurs or advances. Recent advancements in immunotherapy, specifically immune checkpoint inhibitors, have provided a promising therapeutic option for such cases, especially with FDA-approved drugs like pembrolizumab, durvalumab, and dostarlimab. The molecular classification of EC, particularly mismatch repair deficiency, has proven essential in identifying tumors that are likely to respond to immune checkpoint inhibitors, owing to their increased tumor mutational burden and higher PD-L1 expression. However, mismatch repair (MMR) status alone is insufficient to predict immune responses as treatment outcomes are also substantially influenced by tumor microenvironment composition, immune infiltration, and inter-individual variability. Emerging cell therapies like Chimeric Antigen Receptor (CAR) T cells and tumor-infiltrating lymphocytes offer hope for addressing non-immunogenic tumors, overcoming immune evasion mechanisms that limit natural immune responses.

## 1. Introduction

The aim of this review is to examinate the role of mismatch repair deficiency (MMRd) in endometrial cancer and its impact on immunotherapy approaches. We assess the efficacy of immune checkpoint inhibitors in MMRd endometrial cancer, with a particular focus on tumors with different tumor microenvironment features. We also explore emerging therapies, including cell-based approaches, to address resistance in “cold” tumors. This review summarizes current research and emphasizes the need for personalized treatment strategies in endometrial cancer, considering both molecular and immune factors.

### 1.1. Endometrial Cancer

Endometrial cancer (EC) is the most common gynecological malignancy in high-income countries, with its incidence steadily increasing alongside mortality rates. By 2020, EC had become the sixth most common cancer among women globally, with 471,336 new cases and 89,000 deaths reported [1,2,3]. This increase is largely driven by well-established risk factors, including obesity, older age, exposure to endogenous and exogenous estrogen, diabetes, nulliparity, and the use of tamoxifen [4]. Furthermore, epidemiological studies have identified associations between dietary factors, physical activity, and EC [5,6]. This is particularly evident in countries where micronutrients, such as vitamins, are often replaced by high-calorie diets and sedentary lifestyles. Estrogens derived from fats and lipid stores in adipose tissue, or those produced by the adrenal cortex, are known to be associated with EC [7].

Most women are diagnosed at early stages and experience favorable outcomes. However, those with advanced EC face limited treatment options and poor prognoses. Hysterectomy combined with chemotherapy (with or without radiotherapy) remains the standard first-line treatment for advanced disease, while chemotherapy alone (including carboplatin and paclitaxel) represent the gold standard for the management of most metastatic and recurrent EC [8,9].

### 1.2. Histological Type

All endometrial carcinomas are classified in accordance with the fifth edition of the WHO Classification of Tumors of Female Genital Organs. This classification recognizes the following histological subtypes: 1. endometrioid carcinoma, further divided into low-grade (grades 1 and 2) and high-grade (grade 3); 2. serous carcinoma; 3. clear cell carcinoma; 4. mixed carcinoma; 5. undifferentiated carcinoma; 6. carcinosarcoma; 7. rare variants like mesonephric-like carcinoma; and 8. gastrointestinal mucinous-type carcinoma. Each subtype has distinct molecular characteristics, histological features, precursor lesions, and clinical behaviors, and evidence suggests that accurate histological classification may play a crucial role in tumor staging [10,11].

### 1.3. Grading of EC

The grading of endometrioid endometrial carcinomas (EECs) is critical for prognostic evaluation [11,12]. The grading criteria primarily focus on architectural features [13]. Low-grade EECs are subdivided into grade 1 (with up to 5% solid non-glandular growth) and grade 2 (with 6% to 50% solid growth). High-grade EECs (grade 3) are defined by having over 50% solid component. It is important to note that excessive nuclear atypia can raise the grade of a tumor by one level, and in cases of squamous differentiation, grading is based on the glandular component [11]. Grading is particularly important in non-specific molecular-type endometrioid cancers, as mismatch repair deficiency and POLE-mutated types can exhibit mutations that make them appear high-grade [14].

### 1.4. Molecular Classification

Given the limitations of traditional histological classification, in 2013, the Cancer Genome Atlas (TCGA) introduced a molecular classification system [15]. This system divides endometrial cancers into four groups: 1. DNA polymerase epsilon mutated (POLEm); 2. microsatellite instability-high or MMRd; 3. copy number alteration-high/serous TP53 abnormal (somatic copy number alteration-high (SCNA-high)); and 4. SCNA-low [15]. Several studies later advanced a comparable classification system, which is more readily applicable, utilizing the immunohistochemical analysis of tumor protein p53 and mismatch repair proteins (MLH1, PMS2, MSH2, MSH6), along with sequencing to detect mutations in the exonuclease domain of POLE [16]. Each group is defined by distinct molecular characteristics, which in turn influence their prognoses. POLE-ultra-mutated cancers, marked by a high mutational burden, are generally associated with a favorable prognosis. In contrast, tumors with MMR deficiency, stemming from defects in DNA mismatch repair genes, exhibit an intermediate prognosis. Moreover, copy number-high tumors, predominantly serous carcinomas with abnormal p53 (p53abn) expression, are linked to poorer outcomes, while copy number-low tumors, primarily endometrioid carcinomas, tend to show intermediate prognostic profiles [17]. Many professional organizations, including the WHO, European Society of Gynecological Oncology, and FIGO, recommend incorporating molecular subtyping into patient care to improve staging and prognosis [10]. Furthermore, evidence suggests that patients presenting with multiple classifiers, such as MMRd-p53abn, should be assigned to the MMRd category, while those with POLEm-p53abn are best classified under POLEm [15].

### 1.5. Mismatch Repair Deficiency

Before a cell divides, it must replicate its DNA to provide an identical copy for each daughter cell. Accurate DNA replication is crucial for maintaining genome integrity, and this accuracy is controlled by three highly conserved biological processes: Firstly, correct base selection at each position, performed by the polymerase activity of DNA polymerases (ε and δ). Secondly, the proofreading of the newly synthesized strand, carried out by the exonuclease domains of said DNA polymerases. Finally, the post replication correction of DNA mismatches, governed by the MMR system [18,19]. DNA mismatches generally appear during replication and can have two forms: Either base–base mismatches or insertion–deletion errors in repetitive sequences. Repetitive base sequences, known as short tandem repeats or microsatellites, are distributed throughout the genome. Insertion–deletion errors in microsatellites are particularly resistant to detection by the proofreading function of DNA polymerases [20,21]. Alternative phenomena that disrupt proper base pairing, such as spontaneous chemical modifications (deamination and depurination) [22] or exposure to external damage (UV radiation, chemical exposure, and ionizing radiation) will also be repaired by the MMR system [23].

Mismatch repair proteins—MLH1, PMS2, MSH2, and MSH6—identify mismatches and facilitate their excision, followed by accurate resynthesis and ligation. When the mismatch repair system is defective, the cell becomes prone to accumulating mutations, which can lead to cancer development [24,25]. While all types of mismatches accumulate when mismatch repair is defective, microsatellites are especially vulnerable, leading to microsatellite instability, characterized by widespread insertion–deletion (indel) errors in microsatellites [26]. Microsatellite instability (MSI) is a direct consequence of MMRd, and the two terms are often used interchangeably [27]. MMRd can be detected through immunohistochemistry (IHC) for MMR proteins or through MSI testing. Both methods offer similar sensitivity and show about 96% concordance [27]. MMR IHC assesses the presence or absence of the four routinely evaluated MMR proteins (MSH2, MSH6, MLH1, and PMS2) in cancer cells as well as EPCAM, since the deletion of heterozygous sequences at the 3’ end of EPCAM can result in MSH2 inactivation in EPCAM-expressing tissues by causing the promoter hypermethylation of MSH2 [28,29]. MMR IHC is cost-effective, widely accessible, and allows pathologists to correlate results with tissue morphology. MSI testing detects changes in the lengths of selected microsatellites, caused by indel errors, by comparing normal cells to tumor cells on DNA extracted from tumor tissue. Thus, MSI testing is performed by multiplex polymerase chain reaction (PCR) followed by fragment length analysis or through next-generation sequencing (NGS). For PCR assays, several PCR-based automated systems are commercially available and use a set of five to seven microsatellite biomarkers examined on formalin-fixed, paraffin-embedded tissue samples with a fully automated workflow. NGS-based computational detection systems are also available and are based on the comparison of microsatellite length or on calculating the indel border in microsatellites. Given the complementary nature of these methods, MMR IHC is often used first to identify MMR defects, with MSI testing following as a secondary step [30]. For these reasons, from hereon, we will refer to both MSI-High and MMRd, exclusively as MMRd.

## 2. Methods

In order to choose the literature to create this review, we followed the guidelines described in a 2022 paper by Javeed Sukhera [31]. The study selection was conducted using two major databases, PubMed and Scopus, before November 2024. Various combinations of terms, such as “endometrial cancer”, “immunotherapy”, “immune infiltration”, “tumor microenvironment”, and “immunosuppression”, were employed in the search. Given the interdisciplinary nature of this study, a wide range of data sources, encompassing peer-reviewed articles, such as reviews, systematic reviews, and investigative papers, as well as book chapters were considered. Articles were considered eligible for the review if they met the following inclusion criteria: (a) gave relevant information on endometrial cancer; (b) gave relevant information on immunotherapy, tumor microenvironment, or immune checkpoint inhibitors; (c) gave relevant information on endometrial cancer immune-based therapies. Exclusion criteria were as follows: (i) editorials or unpublished studies, (ii) in silico analysis only, (iii) data not fully written in English.

## 3. Immunotherapy for EC Treatment

Around 25–30% EC are characterized by MMRd [32]. In most cases, MMRd occurs sporadically within the endometrium, primarily due to the methylation of the MLH1 promoter region, which leads to the epigenetic silencing of MLH1 [33]. Additionally, germline mutations in MMR genes are a source of MMRd EC [34]. A final smaller subset of MMR-deficient EC (3–5% of all EC cases) arise from Lynch syndrome, a genetically inherited cancer predisposition syndrome [35]. Since the advent of molecular classification for the differentiation of various types of EC, there has been an increasing emphasis on the potential of immunotherapy for patients with MMRd [36,37]. Building upon the promising findings regarding the efficacy of ICIs in individuals with a substantial burden of somatic mutations, researchers have emphasized the heightened sensitivity of MMRd patients to ICI treatment [38]. Several biological factors have been studied and proposed as contributors of sensitivity to immunotherapy [39]. One key aspect is that MMRd-induced genomic instability leads to a higher TMB, which results in the production of abnormal neoantigens, increasing tumoral immunogenicity [40,41]. However, this view remains a topic of debate, as recent studies have shown that MMRd TMB may not be enough to elicit immunogenicity per se and that it is rather specific clonal neoantigen burden which elicits responses to immunotherapy [42,43]. Moreover, tumors that present low immunogenicity or immunosuppressive tumor microenvironments will fail to respond properly to immunotherapy [44]. In the following pages, we will review the biological mechanisms by which immunotherapy is effective against certain endometrial carcinomas, and we will provide a detailed picture of currently FDA-approved immunotherapies and currently on-going clinical trials.

### 3.1. PD-1/PD-L1 Expression

Programed cell death protein 1 (PD-1) is a cell surface protein classified as a membrane protein in the immunoglobulin superfamily in humans. It plays a key role in suppressing both adaptive and innate immune responses [45]. PD-1 is expressed on various immune cells, including activated T cells, natural killer cells, B lymphocytes, macrophages, dendritic cells, or monocytes and restrains immune responses through inhibitory signaling in T cells and regulatory T cells (Tregs) [46,47]. Transcription factors such as nuclear factor of activated T cells (NFAT) and forkhead box protein O1 (FOXO1) can initiate the transcription of PD-1 [48]. Tumor cells and immune cells in the tumor microenvironment increase programmed death ligand 1 (PD-L1) expression to evade immune lysis by binding PD-L1 protein to the PD-1 receptor on T cells [49,50], inhibiting their function and preventing immune-mediated cancer elimination [51]. As previously explained, MMR status is considered a key factor in ICI success, mainly due to the increased recruitment of PD-L1 cells [52]. In EC, PD-1 can be found in approximately 60–65% of cases and PD-L1 in about 25–70% of cases [53]. This variability can be attributed to differences in study methodologies, antibody clones used for detection, and even cut-off thresholds [54]. Discussion continues regarding the relevance of PD-L1 as a prognostic factor and/or therapeutic opportunity since expression across different molecular subtypes of EC is very heterogeneous [55].

Multiple studies have highlighted a distinction between cases with detectable PD-L1 expression and those lacking it entirely (alternatively known as “hot” and “cold” tumors) [56] and proposed PD-L1-positive EC tumors as good candidates for ICI [57]. The therapeutic relevance of ICIs in PD-L1-positive tumors is once again highlighted by the fact that these are able to recruit tumor associated CD3+ and CD8+ lymphocytes, regardless of MMR status [58,59]. Furthermore, PD-L1 expression has also been linked to the increased concentration of Th1 cytokine, chemical mediators, and interferons, alongside distinct gene expression patterns [60,61]. Although the interplay of PD-L1 and immune infiltration has not been deeply studied in the context of EC yet [51], in other gynecological cancers such as ovarian cancer, research shows that the pro-inflammatory cytokine interferon-gamma (IFN-γ) can directly enhance PD-L1 expression, further contributing to tumor progression [62,63]. In this sense, PD-L1 expression levels have been correlated with vascular and myometrial invasion in EC [59], which opens the possibility of using PD-L1 as a prognostic factor, as well. These data are backed by other studies where it has been seen that PD-L1-positive endometrial tumors exhibit significantly higher levels of invasiveness and lymph node metastasis compared to the PD-L1-negative group. However, despite these aggressive features, the authors were unable to demonstrate significant differences in overall survival or recurrence rates [57]. The complex interplay between MMRd status and PD-L1 expression has also been studied by the European Society for Medical Oncology in an attempt to integrate data from PD-L1 expression, MMR status, and tumor burden [64]. PD-L1 and PD-1 inhibitors are spearheading the ICI approach in cancer treatment [65,66]. These inhibitors work by binding to receptors like PD1 and PD-L1 to prevent the interaction between PD-1 and PD-L1, preventing tumor cells from silencing immune response performed by T lymphocytes, thus effectively reactivating antitumor response [67] through the following canonical apoptosis pathways: (A) Perforin/Granzyme Pathway: Cytotoxic granules released by CTLs induce caspase-mediated apoptosis in tumor cells [68]. (B) Death Receptor Pathways: CD8+ cells upregulate FasL and TRAIL, binding to their respective receptors on EC cells and triggering extrinsic apoptosis [69]. As previously stated, the upregulation of PD-L1 can contribute to the inhibition of the active T-cell immune surveillance of tumors through the inhibition of T cell proliferation and cytokine production [70].

**Figure 1:**
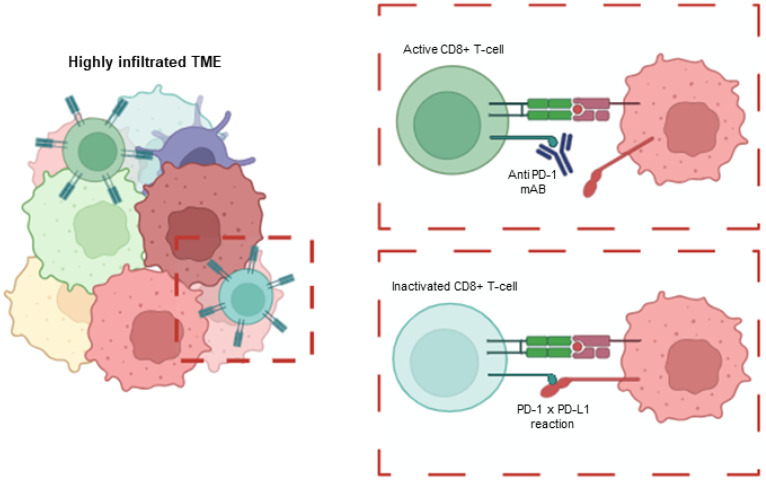
Mechanism of PD-1-mediated immune suppression and the action of AntiPD-1 therapy. MMRd-deficient tumors are more prone to accumulate a high tumor mutational burden, which leads to the generation of neoantigens that can elicit an immune response against the tumor. However, tumor cells often suppress this immune activation by high jacking the PD-1/PD-L1 immune checkpoint pathway. The use of PD-1 inhibitors, such as pembrolizumab, blocks the interaction between tumor cells and immune CD8+ t cells, effectively restoring the immune system’s ability to recognize and attack tumor cells. Created with Biorender.com.

Thus, blocking PD-1 activity results in decreased tumor growth in syngeneic mouse models and patients [70,71]. Based on these data, there are now three approved therapies that target PD-L1 or PD-1 in EC: pembrolizumab, durvalumab and dostarlimab-gxly. See Figure 1 for summarized information.

#### 3.1.1. Pembrolizumab

Pembrolizumab (KEYTRUDA, Merck) is a monoclonal antibody that specifically binds to the PD-1 receptor, inhibiting its interaction with PD-L1 and PD-L2. This blockade effectively disrupts the PD-1 pathway-mediated suppression of the immune response, thereby enhancing the anti-tumor immune response [71]. Pembrolizumab is FDA-approved for the treatment of 17 types of cancer, including advanced EC [72]. There are three different approved indications of use for pembrolizumab in EC: either as standalone therapy or in combination with other therapies. As a standalone therapy, pebrolizumab has been approved to treat patients with unresectable or metastatic dMMR EC. The results of KEYNOTE-158 (NCT2628067) showed an objective response rate of 12% for complete responses and 33% for partial responses. But, more importantly, the duration of response exceeded 12 months in 68% of patients and more than 24 months in 44% of patients (Table 1) [73]. In this trial, 64.8% of all patients faced treatment-related adverse events, of which 14.6% were considered severe and one developed pneumonia. More interestingly, immune-mediated adverse events (regardless of attribution to treatment) occurred in 23.2% of patients. Finally, 5.2% of patients discontinued treatment because of an immune-mediated adverse event, although none were severe.

While these results are conclusive in regards of pembrolizumab efficacy, its combination with first-line treatment therapies has proven to be more beneficial. In the KEYNOTE-868/NRG-GY018 (NCT03914612), the use of pembrolizumab in combination with paclitaxel and carboplatin significantly improved the overall survival of all patients regardless of MMR status (Table 1). In regards of safety, in the MMRd cohort, 3 of 215 patients died during the clinical trial. These deaths included a variety of severe events, including cardiac arrest, sepsis, and lower gastrointestinal hemorrhage. One of these patients was in the pembrolizumab group and two were in the placebo group; an association with the trial group was considered to be unlikely by the treating physician. In the MMRp cohort, eight patients died (six in the pembrolizumab group and two in the placebo group), which included sepsis in four patients, cardiac arrest in two patientsm, and small intestinal obstruction or sudden death not otherwise specified in one patient each. Grade 5 cardiac arrest was deemed to be possibly related to pembrolizumab in one patient in the MMRp cohort [74].

This is not the only combined use that has been approved for pembrolizumab. Lenvatinib mesylate (LENVIMA, Eisai, Tokyo, Japan) is a multi-targeted kinase inhibitor that primarily inhibits the kinase activities of vascular endothelial growth factor receptors (VEGFRs), specifically VEGFR1, VEGFR2, and VEGFR3 [75]. These kinases play key roles in tumor angiogenesis, growth, and immune regulation [76]. In syngeneic mouse tumor models, Lenvatinib reduced the presence of tumor-associated macrophages and increased the population of activated cytotoxic T cells. The combined use of pembrolizumab with Lenvatinib mesylate has proven to be more effective than traditional chemotherapy [77]. In the KEYNOTE-775 trial (NCT03517449), pembrolizumab was administered in combination with Lenvatinib in patients with advanced EC who had previously received platinum-based chemotherapy, regardless of MMR status. The overall survival, progression-free survival, objective response rate, and duration of response were significantly higher in the pembrolizumab + Lenvatinib group when compared to the group treated with doxorubicin or paclitaxel (Table 1) [78]. Regarding safety, both Lenvatinib + pembrolizumab (97.3%) and chemotherapy (93.8%) cohorts experienced adverse effects of any grades. The most common and differential adverse effects the first group experienced were hypertension, hypothyroidism, diarrhea, decreased appetite, proteinuria, and weight loss. All these affections were seen at both mild and severe levels. These results highlight the relevance of the tumor microenvironment modulation in therapy response extending considerations beyond MMR status alone.

#### 3.1.2. Duravlumab

Durvalumab (IMFINZI, Astrazeneca, Cambridge, UK) is a human monoclonal antibody of the IgG1 kappa isotype that targets PD-L1, preventing its interaction with PD-1 and CD80. In preclinical studies, PD-L1 blockade with durvalumab enhanced T-cell activation in vitro and led to significant reductions in tumor size in mouse models co-engrafted with human tumors and immune cells [79]. Durvalumab has not been approved as a standalone therapy for EC and can only be used in combination with carboplatin–paclitaxel followed by maintenance durvalumab with Olaparib, a flagship PARP inhibitor that prevents DNA damage reparation [80,81]. Durvalumab interacts with carboplatin–paclitaxel and Olaparib through complementary mechanisms that enhance antitumor efficacy. Carboplatin and paclitaxel act by inducing DNA damage and mitotic arrest, leading to apoptosis. Carboplatin forms DNA crosslinks that disrupt replication and transcription, while paclitaxel stabilizes microtubules, halting cell division in the G2/M phase. Furthermore, chemotherapy-induced DNA damage activates the cGAS-STING pathway, resulting in type I interferon production, which further amplifies T-cell recruitment and activation [82]. Durvalumab complements this effect by blocking immune evasion mechanisms. Tumor cells often upregulate PD-L1 in response to DNA damage [83], suppressing T-cell activation. Durvalumab counteracts this suppression, restoring immune system activity and enabling CD8+ T cells to recognize and destroy tumor cells. Olaparib enhances this synergy by exploiting the synthetic lethality of tumors deficient in DNA repair pathways. PARP inhibition leads to the accumulation of DNA single-strand breaks, which progress to lethal double-strand breaks in tumors with deficient homologous recombination repair, such as those with MMR deficiency [84]. These DNA lesions further increase tumoral immunogenicity, creating a precondition for the heightened efficacy of durvalumab.

The use of these inhibitors is a promising strategy for targeting cancers with DNA damage, such as MMRd EC [85]. In the DUO-E trial (NCT04269200), durvalumab was used in combination with carboplatin and paclitaxel followed by durvalumab monotherapy with or without Olaparib for adult patients with primary advanced or recurrent dMMR EC. Progression-free survival, response to treatment, and duration of response were significantly higher in the durvalumab + Olaparib group when compared to durvalumab monotherapy and placebo groups (Table 2). In this trial, the most commonly observed adverse effects of any grade included anemia, nausea, fatigue or asthenia, and alopecia, either during the treatment or maintenance phase. Albeit, most adverse effects occurring during the maintenance phase were low grade. In the control, durvalumab, and durvalumab + olaparib arms, 56.4%, 54.9%, and 67.2% of patients experienced adverse effects, and the incidence in the maintenance phase was 16.6%, 16.4%, and 41.1%, respectively. The overall incidence of grade 3 or higher neutropenia was 23.3%, 21.7%, and 26.9%, respectively, and the overall incidence of grade 3 or higher anemia was 14.4%, 15.7%, and 23.5%, respectively. More importantly, fatal events occurred in 3.4%, 1.7%, and 2.1%, respectively [86].

#### 3.1.3. Dostarlimab-Glxy

Dostarlimab-glxy (JEMPERLI, GlaxoSmithKline, Brentford, UK) is a humanized monoclonal antibody of the IgG4 isotype that targets the PD-1 receptor. In preclinical syngeneic mouse tumor models, the inhibition of PD-1 activity has shown to reduce tumor growth, indicating its potential to enhance anti-tumor immunity and slow solid tumor progression, regardless of their MMR state [87]. Similarly to pembrolizumab, dostarlimab has been approved as a single agent and in combination with carboplatin to treat EC. In the GARNET study (NCT02715284), dostarlimab was used as a single agent in patients with dMMR advanced EC that had been unsuccessfully treated with any platinum-based regime. The objective response rate was 15.6% complete response and 29.8% partial response. The duration of response was over 12 months for 85.9% of patients and over 24 months for 54.7% (Table 3). In the combined treatment cohort, the most common adverse effects of any grade were fatigue (17.6%), diarrhea (13.8%), and nausea (13.8%). The most common grade ≥ 3 TRAEs were anemia (2.8%), alanine aminotransferase increased (1.4%), diarrhea (1.4%), fatigue (1.4%), amylase increased (1.4%), and lipase increased (1.4%) for the combined patient population. More importantly, no deaths were attributable to dostarlimab; however, there were 16 (5.5%) discontinuations due to adverse effects. The most common TRAEs leading to discontinuation were the alanine aminotransferase increase (1.0%), aspartate transaminase increase (0.7%), and transaminase increase (0.7%) [88]. In the RUBY trial (NCT03981796), dostarlimab was used in combination with carboplatin–paclitaxel, significantly increasing progression-free survival among patients with primary, advanced, or recurrent EC, regardless of MMR status. Nevertheless, the most substantial therapeutic benefits were seen in dMMR patients (Table 3). The most common adverse effects were nausea (53.9% of the patients in the dostarlimab group and 45.9% of those in the placebo group) and fatigue (51.9% and 54.5%), and alopecia (53.5% and 50.0%) was observed as well. Interestingly, the incidences of grade 3 or higher adverse events and serious adverse events that occurred or worsened during treatment were each approximately 10 percentage points higher in the dostarlimab group than in the placebo group (adverse events, 70.5% vs. 59.8%; serious adverse events, 37.8% vs. 27.6%). Consequently, the discontinuation of dostarlimab or placebo because of adverse events occurred in 17.4% of patients in the dostarlimab group and in 9.3% of patients in the placebo group. The most common adverse events leading to the discontinuation of dostarlimab or placebo were maculopapular rash and infusion-related reaction (1.2% each) in the dostarlimab group and thrombocytopenia (1.2%) in the placebo group. Similarly to the adverse effects observed in all other clinical trials, the most common immune-related adverse events were hypothyroidism (11.2% of the patients in the dostarlimab group and 2.8% of those in the placebo group), rash (6.6% and 2.0%), arthralgia (5.8% and 6.5%), and an increase in alanine aminotransferase levels (5.8% and 0.8%). Finally, five deaths due to adverse events occurred in the dostarlimab group, and no deaths occurred in the placebo group. Dostarlimab-related deaths included myelosuppression, hypovolemic shock, and three were judged not to be related to the dostarlimab regimen [89].

### 3.2. Immune Infiltration in Tumor Microenvironment

The tumor microenvironment (TME) plays a significant role in EC progression, and it is composed of diverse elements, including immune cells, fibroblasts, pericytes, endothelial cells, mesenchymal stroma, and the surrounding extracellular matrix components [90,91,92]. Stromal cells significantly influence tumor metabolism and the evasion of the immune system. Immunosuppression in the TME promotes tumor development by recruiting inhibitory immune cells, such as CD4+ T cells (Tregs) and dendritic cells, to evade immune surveillance; this immune cell infiltration, influenced by various factors, is closely linked to tumor growth and invasion [93,94]. Tumors and even different regions within the same tumor often show significant heterogeneity in the immune cell infiltration component, which contributes to the variability in therapeutic responses [59]. This heterogeneity is evident in the varying composition of T-lymphocyte subsets, B-cells, and macrophages within and across tumor microenvironments. Intratumoral immune cell variability gives rise to three different cancer-immune phenotypes: inflamed, excluded, and desert [95]. For example, regulatory Tregs deploy multiple suppressive mechanisms to dampen pro-inflammatory responses within the tumor, effectively altering ICI therapeutic success [96]. This variability extends to metastases, which also display immunogenic differences [97,98]. MMR status has been proven to influence tumor immune cell infiltration multiple times [99]. MMRd tumors have a higher concentration of CD8+ T cells as well as PD-L1+ cells when compared to MMRp counterparts [100]. These changes in the cellular TME increase overall immunogenicity, allowing ICI therapeutic success [100,101].

However, direct correlation between MMR status and immune infiltration is still a matter of discussion. In fact, MMRd non-responder patient cohorts completely lack immune infiltration, and, more strikingly, no inverse correlation with PD-L1 expression is seen either [102]. These data suggest that other factors that are not yet considered may influence in tumor infiltration, together with MMR status or PD-L1 expression. Moreover, it indicates that tumor immune infiltration may be a better prognostic tool than MMR or PD-L1 expression and elucidates the variation observed in therapy response to ICIs [103,104]. Multiple papers support this notion from a variety of standpoints. The general trend supports that specific clonal mutation burden, rather than MMR status or TMB, are more likely to correlate with immune infiltration and, thus, therapeutic success [42]. Indeed, it has been proven how concrete immune-related gene signatures are able to correlate with immune infiltration, pathological characteristics [105], and prognoses [106] better than MMR status. Overall, these studies demonstrate the potential clinical utility of immune profiling in EC, offering a tool for improved prognosis and individualized treatment strategies. These examples highlight the growing use of immune-based scoring systems and the relevance of immune TME in therapeutic success, with ongoing research further refining and developing similar approaches [107,108]. (See Figure 1 for summarized information).

#### Genetic Relevance in the Immunosuppressive TME

As previously explained, such differences in TME immune infiltration can be accounted to specific mutational burden. The flagship example is that of the phosphatase and tensin homolog (PTEN), the most frequently mutated tumor suppressor gene in EC [109], which has also been associated with the development of an immunosuppressive TME across multiple cancers [110,111] PTEN deficiency impairs key immune signaling pathways, particularly the interferon (IFN) pathway, through the serine dephosphorylation of regulatory transcription factor 3 (IRF3) to enable its own nuclear migration [112,113]. IFN is expected to boost the recruitment and activation of antitumor T cells and natural killer (NK) cells, while also promoting antigen presentation by dendritic cells [114]. Indeed, studies in various types of cancer show that the hyperactivation of the type I IFN response is strongly associated with better outcomes in immunotherapy, as it increases immune cell infiltration and overall antigen presentation by tumor cells [115]. Consequently, PTEN depletion disrupts the activation of immune cells like dendritic cells, T lymphocytes, and NK cells across multitude of cancers [116]. This results in a TME dominated by Tregs, myeloid-derived suppressor cells [117], and immunosuppressive M2-like macrophages, all of which suppress the body’s antitumor response Tregs, specifically have been a focus of study and have been proven to be of great relevance in the development of an immunosuppressive TME [118,119]. In fact, their abundance in the TME is often associated with poor patient outcomes and to resistance to PD-1/PD-L1-targeted therapies across various cancer types, which indicates that EC would behave similarly [120,121]. Following this rationale, it has been proven that the combination treatment of anti-PD-L1 with anti-TGFβ restores immune infiltrating capacity and rescues tumor sensitivity to ICI, providing strong evidence that PTEN depletion causes immunotherapy resistance through TME modification [91].

## 4. On-Going Clinical Trials

### 4.1. New Indications for Already Approved Drugs

Following the positive outcomes observed with ICIs in combination in second-line treatments, phase III randomized clinical trials are now assessing ICIs as a single agent in the first-line setting. These trials aim to explore chemotherapy-free alternatives for patients with newly diagnosed or recurrent advanced endometrial cancer. Specifically, the NCT05173987 and NCT05201547 trials are investigating pembrolizumab and dostarlimab, respectively, versus chemotherapy in patients with dMMR advanced or relapsed endometrial cancer. Likewise, there are multiple on-going clinical trials that explore new combinations of already approved drugs. Dostarlimab has two examples (NCT05819892 and NCT05559879): The combination of dostrarlimab with chemoradiation in patients with stage IIIC EC and the combination of dostarlimab with a targeted inhibitor of VEGF, cabozantinib. At the time of writing this article, there are five ongoing clinical trials focused on novel indications for pembrolizumab. Such are the cases of NCT03835819 and NCT05572684, where they test pembrolizumab in combination with Mirvetuximab soravtansine in FRα-expressing endometrial cancer and NC410 in advanced or metastatic solid tumors with MMRp status. Other clinical trials keep working on assessing efficacy of pembrolizumab combined with standard chemotherapy (paclitaxel and carboplatin) as well as clinical outcomes for stage III-IV or recurrent endometrial cancer. Pembrolizumab with radiation therapy (NCT04214067) is being studied as well. There is a final clinical trial, NCT05269381, that explores the safety and tolerability of a personalized neoantigen peptide-based vaccine combined with pembrolizumab in patients with advanced solid tumors (See Table 4 for additional information).

### 4.2. New Monoclonal Antibodies

Other studies focus on novel monoclonal antibodies, either already approved for other cancers or as completely new agents. Nivolumab is already approved for the treatment of melanoma, non-small cell lung cancer, malignant pleural mesothelioma, renal cell carcinoma, classical Hodgkin lymphoma, squamous cell carcinoma of the head and neck, urothelial carcinoma, dMMR metastatic colorectal cancer, hepatocellular carcinoma, esophageal cancer, gastric cancer, and gastroesophageal junction cancer. Now it is being studied in various clinical trials. NCT05112601 studies Nivolumab with or without ipilumumab in dMMR-recurrent EC. There are other clinical trials that explore the therapeutic possibilities of the precise combination of NCT03508570 and NCT02834013. The first is aimed at treating patients with recurrent or high-grade gynecologic cancer with metastatic peritoneal carcinomatosis, while the second is targeting patients with rare tumors. Finally, in NCT04278144, researchers are studying a novel antibody known as BDC-1001, as a single agent or in combination with Nivolumab in advanced HER2-expressing solid tumors. Avelumab, an anti-human CD274 antibody already used in merkel cell carcinoma, bladder, and urinary tract cancer, is being tested as well. In NCT06518564, researchers are testing the safety and efficacy of Avelumab in combination with M1774, a type of ATR inhibitor for ARID1A-deficient EC. Another great example is the use of Sacituzumab, widely used for very challenging diseases such as triple-negative breast cancer, HR+/HER2− breast cancer, and various cancers of the urinary tract. It is currently being studied against the treatment of the physician’s choice in participants with EC following platinum-based chemotherapy and immunotherapy (NCT06486441).

Retifanlimab (NCT04463771) is another antibody that is currently under clinical trials. There are also multiple studies that aim to prove the efficacy of antibodies never approved before. In NCT05592626, researchers aim to a selective T cell receptor (TCR) known as STAR0602 in participants with advanced solid tumors as monotherapy or in combination with other immunotherapy or targeted agents. In NCT04272034, researchers aim to test the tolerability, pharmacokinetics, and pharmacodynamics of INCB099318 in participants with advanced solid tumors (See Table 4 for additional information).

#### Cell Therapies

There is a final group of clinical trials that focus on applying cell therapies in EC. In NCT06481592, researchers aim to study the efficacy and safety of Lifileucel (tumor-infiltrating lymphocytes) in advanced EC patients who have previously received treatment with platinum-based chemotherapy and an anti-PD-1/PD-L1 agent in a recurrent or advanced setting, either sequentially or in combination. NCT01174121 also involves tumor-infiltrating lymphocytes but undertakes a rather experimental approach. Here, researchers from the NCI surgery branch will extract lymphocytes from patients’ tumors and expand them in vitro to elicit a large-scale tumor infiltration. This technique, already used in melanoma patients, will be tested in endometrial cancers. An alternative approach is the direct activation of immune effector cells (CD8+ T- and NK cells) while avoiding the activation of immunosuppressive Tregs. Finally, numerous studies focus on Chimeric Antigen Receptor (CAR) T cell therapy. NCT05194735 studies autologous T cells to express T cell receptors in various solid tumors. In NCT04660929, researchers harness the power of CAR-macrophages for the treatment of HER-2-overexpressing solid tumors. Similarly, in NCT04319757, researchers are testing ACE1702 in subjects with advanced or metastatic HER2-expresing solid tumors (See Table 4 for additional information).

## 5. New Strategies: Pre-Clinical Data

In a paper from 2019, Wang and his colleagues aimed to understand how CD8+ T cells induce cell death in tumor cells during immunotherapy. Their results showed that PD-L1 blockade therapy induced ferroptosis, a type of cell death caused by iron-dependent lipid peroxidation. Specifically, CD8+ T cells were found to induce ferroptosis in cancer cells by releasing IFN-γ during immunotherapy with anti PD-L1. IFN-γ inhibited the uptake of cystine, a crucial molecule for antioxidant production in tumor cells, leading to increased oxidative stress and ferroptotic cell death [122]. This mechanism suggests that CD8+ T cells not only kill tumor cells through direct cytotoxic activity but also regulate ferroptosis, enhancing the efficacy of cancer immunotherapies. This link offers new therapeutic insights for improving immune-based treatments by combining them with ferroptosis-inducing drugs. It has been known for a number of years that cancer cells that undergo an epithelial to mesenchymal transition (EMT) are more susceptible to ferroptosis induction on account of their lipidome [123]. In this context, immunotherapy appears as a therapeutic opportunity for those cancers that are more plastic and have multiple resistances and increased migratory capacities.

## 6. Concluding Remarks

EC is typically a highly treatable malignancy in its early stages; however, therapeutic options for advanced or recurrent cases remain scarce [5,9]. Over recent years, immunotherapy has emerged as a promising treatment avenue for EC due to the immunogenic nature of these tumors. Immunogenic tumors exhibit characteristics that make them more detectable to the immune system, which allows for the potential ICIs to be harnessed [66,98]. Several ICIs, including pembrolizumab, durvalumab, and dostarlimab, are currently FDA-approved for use in EC, and more importantly, many more are already being trialed. Their introduction has shown marked improvements in clinical outcomes compared to conventional chemotherapy, signaling a paradigm shift in EC treatment approaches [79,84,85].

In order to extract their maximum therapeutic power, MMR status is currently being used as a standard for both prognosis and treatment response evaluation in EC [8,94]. MMR deficiency leads to MSI-High, resulting in a high TMB that increases the likelihood of neoantigen formation [27]. This makes the TME richer in immune cells (immunogenic) or “hot”. However, MMRd cancer cells often show higher PD-L1 expression due to chronic inflammatory signaling, which allows these tumors to evade immune surveillance via the PD-1/PD-L1 axis [70]. At the same time, it makes them prime candidates for treatment with PD-1 inhibitors, which reactivate immune response. In fact, MMRd status in EC tends to correlate with better responses to immunotherapy but poorer overall prognosis if left untreated due to its association with more aggressive tumor phenotypes [37,55,69]. The interplay between MMR status, TMB, immune infiltration, and PD-1/PD-L1 expression is critical to understand immunotherapy responses. As previously said, under normal circumstances, MMRd EC tumors are more responsive to ICIs such as pembrolizumab due to this heightened immune recognition [38]. However, some MMRd tumors that have high TMB and high PD-1 expression still show poor immune infiltration (“cold” tumors) and will not respond favorably to ICIs [40,116]. All these data confirm two important aspects of tumor immune biology: first, what really matters in ICI therapy success is the TME immune cell composition, and, second, that it could vary independently from MMR status and TMB. Indeed, multiple studies aimed and succeeded in generating specific immune-based gene signatures that have substantial prognosis value, indicating that specific clonal mutations have much more weight in overall immune infiltration [100]. Some hallmark genes in the construction of a “hot” TME are as follows: INFγ, which recruits CD8+ infiltrating T cells, or STAT1, a key mediator of I INFγ signaling [50,64,107]. On the other hand, PTEN depletion has been shown to generate “cold” TME via the downregulation of IFN production, the subsequent increase in the concentration of Treg cells, and the increased expression of immunosuppressive cytokines, which results in decreased T-cell infiltration in tumors. Strikingly, this phenotype can be reversed with anti-TGFβ treatment [103,104,115,116]. Ultimately, these data suggest that profiling the immune infiltration of the TME is a more reliable tool for determining therapeutic strategies than relying solely on MMR status or PD-L1 expression.

Additionally, one can expect immune cell intra-tumoral variability, observing substantial differences in immune cell infiltration, distribution, and activity across different regions of a single tumor. This variability leads to the emergence of three main cancer-immune phenotypes: inflamed, excluded, and desert [89]. Inflamed sections are generally the most responsive to ICIs, as they are already rich in immune cells. On the other hand, excluded and desert sections typically show minimal immune infiltration, requiring combination therapies that modify the tumor microenvironment to enhance immune cell entry and activity. Tailoring therapy to target both the tumor cells and the immune environment ensures a more comprehensive and effective approach to cancer treatment [43,93]. Failure to address immune heterogeneity within tumors may lead to an initial reduction in tumor size with ICIs, but can promote the survival and expansion of resistant tumor clones, worsening disease progression in the long term. This highlights the importance of personalized treatment approaches that consider the immune landscape of each tumor to optimize outcomes and avoid therapeutic resistance [78]. For these reasons, novel cell-based therapies are already being developed and trialed for advanced and recurrent EC. CAR-T cells and TILs are considered promising treatment options for “cold” and heterogeneous tumors because they provide external immune cells that can directly target and kill cancer cells, bypassing the need for natural immune infiltration. CAR-T cells are engineered to work on cancer-specific antigens independently of MHC proteins. This enables them to recognize and attack cancer cells independently of the natural TME. In contrast, TIL therapy involves extracting a patient’s immune cells from within their tumor, expanding them ex vivo and reinfusing them into the patient. These TILs are highly effective in “cold” tumors because they are already primed to identify and attack cancer cells.

In conclusion, while immunotherapy has been shown as a significant promise in the treatment of advanced and recurrent EC, its success is heavily dependent on the TME. MMR status, TMB, and immune cell infiltration play key roles in predicting treatment outcomes; however, it is the TME composition that ultimately drives the response to ICIs. Novel cell-based therapies, including CAR-T cells and TILs, hold potential for addressing immune-infiltration issues in “cold” tumors. However, a major challenge remains in expanding these advancements globally as clinical trials in developing countries are limited, primarily due to the lack of comprehensive genetic screening for PD-L1 and PD-1. Future research must focus on refining immune profiling and therapeutic personalization to improve clinical outcomes and address immune heterogeneity in the TME, particularly in more resistant tumor phenotypes.

## Figures and Tables

**Table 1:** Current FDA-approved indications for pembrolizumab in EC. Clinical trial code, indication, experiment design, and key metrics are specified. N.S: Not specified.

Clinical Trial	Indication	Overall Survival (Median in Months)	Progression-Free Survival (Median in Months)	Objective Response Rate % (Complete Response/Partial Response)	Duration of Response (Median in Months)
KEYNOTE-158 (NCT2628067)	Used alone in patients with unresectable or metastatic MSI-H or dMMR endometrial carcinoma	N.S	N.S	12%, 33%	>12 months: 68% >24 months: 44%
KEYNOTE-868/NRG-GY018 (NCT03914612)	In combination with paclitaxel and carboplatin in patients with advanced or recurrent endometrial carcinoma (PMMR and DMMR)	N.S	DMMR population: Pembrolizumab with Placitaxel and carboplatin: NR Placebo with Placitaxel and carboplatin: 6.5 PMMR population: Pembrolizumab with Placitaxel and carboplatin: 11.1 Placebo with Placitaxel and carboplatin: 8.5	N.S	N.S
KEYNOTE-775/STUDY 309 (NCT03517449)	In combination with Lenvatinib in patients with advanced endometrial carcinoma who had been previously treated with at least one prior platinum-based chemotherapy regimen in any setting, including in the neoadjuvant and adjuvant settings (PMMR or not MSI-H)	Pembrolizumab + Lenvatinib: 17.4 months Doxorubicin or paclitaxel: 12.0 months	Pembrolizumab + Lenvatinib: 6.6 months Doxorubicin or paclitaxel: 3.8 months	Pembrolizumab + Lenvatinib: 5%, 25% Doxorubicin or paclitaxel: 3%, 13%	Pembrolizumab + Lenvatinib: 9.2 months Doxorubicin or paclitaxel: 5.7 months

**Table 2:** Current FDA-approved indications for durvalumab in EC. Clinical trial code, indication, experiment design, and key metrics are specified. N.S: Not specified.

Clinical Trial	Indication	Overall Survival (Median in Months)	Progression-Free Survival (Median in Months)	Objective Response Rate % (Complete Response/Partial Response)	Duration of Response (Median in Months)
DUO-E (NCT04269200)	In combination with carboplatin and paclitaxel followed by durvalumab monotherapy for adult patients with primary advanced or recurrent DMMR EC.	N.S	32.6	71.4 (28.6/42.9)	2.4+, 26.9+

**Table 3:** Current FDA-approved indications for dostarlimab in EC. Clinical trial code, indication, experiment design, and key metrics are specified. N.S: Not specified.

Clinical Trial	Indication	Overall Survival (Median in Months)	Progression-Free Survival (Median in Months)	Objective Response Rate % (Complete Response/Partial Response)	Duration of Response (Median in Months)
RUBY (NCT03981796)	In combination with carboplatin and paclitaxel for the treatment of primary advanced or recurrent endometrial cancer.	Overall Population: Jemperli + carboplatin + paclitaxel: 44.6 Placebo + carboplatin + paclitaxel: 28.2 DMMR Jemperli + carboplatin + paclitaxel: NR Placebo + carboplatin + paclitaxel: 30.8	Overall Population: Jemperli + carboplatin + paclitaxel: 11.8 Placebo + carboplatin + paclitaxel: 7.9 DMMR Jemperli + carboplatin + paclitaxel: 30.3 Placebo + carboplatin + paclitaxel: 7.7	Overall Population: Jemperli + carboplatin + paclitaxel: 1(20%, 48%) Placebo + carboplatin + paclitaxel: (12%, 45%) DMMR Jemperli + carboplatin + paclitaxel: (26%, 48%) Placebo + carboplatin + paclitaxel: (11%, 51%)	Overall Population: Jemperli + carboplatin + paclitaxel: 10.8 Placebo + carboplatin + paclitaxel: 6.4 DMMR Jemperli + carboplatin + paclitaxel: N.R Placebo + carboplatin + paclitaxel: 5.4
GARNET (NCT02715284)	As a single agent, used in patients with advanced solid tumors. Patients with dMMR recurrent or advanced EC who had progressed on or after treatment with a platinum-containing regimen.	N.S	N.S	15.6%, 29.8%	Duration > 12 months: 85.9% Duration > 24 months: 54.7%

**Table 4:** Summary of all currently on-going clinical trials based on immune-therapies for endometrial cancer.

NCT	Study Title	Study Status	Sponsor	Collaborators	Locations
NCT06518564	Avelumab and M1774 in ARID1A-mutated Endometrial Cancer (Prior IO)	NOT_YET_RECRUITING	Panagiotis Konstantinopoulos, MD, PhD	EMD Serono|The Applebaum Foundation	Dana-Farber Cancer Institute, Boston, MA, USA
NCT06486441	Study of Sacituzumab Govitecan Versus Treatment of Physician’s Choice in Participants with Endometrial Cancer After Platinum-Based Chemotherapy and Immunotherapy (ASCENT-GYN-01/GOG-3104/ENGOT-en26)	RECRUITING	Gilead Sciences	GOG Foundation|European Network of Gynecological Oncological Trial Groups (ENGOT)	Women’s Cancer Care, Covington, LA, USA,
NCT06481592	A Study of Lifileucel (tumor-infiltrating Lymphocytes) in Adults with Advanced Endometrial Cancer	RECRUITING	Iovance Biotherapeutics, Inc. (San Carlos, CA, USA)		UofL Health—Brown Cancer Center, Louisville, KY, USA
NCT06349642	Predicting Response to Immune Checkpoint Inhibitors Across Solid Tumors Using a Live Tumor Diagnostic Platform	RECRUITING	Mayo Clinic	Elephas Biosciences Corporation	Mayo Clinic in Florida, Jacksonville, FL, 32224, USA
NCT06253494	Pembrolizumab, Lenvatinib and IL-15 Superagonist N-803 in Combination with HER2 Targeting Autologous Dendritic Cell (AdHER2DC) Vaccine in Participants with Advanced or Metastatic Endometrial Cancer	RECRUITING	National Cancer Institute (NCI)		National Institutes of Health Clinical Center, Bethesda, MD, USA
NCT06132958	Sacituzumab Tirumotecan (MK-2870) in Post Platinum and Post Immunotherapy Endometrial Cancer (MK-2870-005)	RECRUITING	Merck Sharp & Dohme LLC	European Network for Gynaecological Oncological Trial groups (ENGOT)|GOG Foundation	MultiCentric
NCT05891197	A Biomarker Screening Protocol for Participants with Solid Tumors	RECRUITING	Lyell Immunopharma, Inc. (South San Francisco, CA, USA)	ICON plc	Accellacare, Rocky Mount, NC, 27804, USA
NCT05819892	Phase I Trial Testing the Safety and Tolerability of Chemoradiation Followed by Chemotherapy + Dostarlimab for Stage IIIC, Node Positive, Endometrial Cancer	RECRUITING	M.D. Anderson Cancer Center	GlaxoSmithKline	M D Anderson Cancer Center, Houston, TX, 77030, USA
NCT05592626	A Study of a Selective T Cell Receptor (TCR) Targeting, Bifunctional Antibody-fusion Molecule STAR0602 in Participants with Advanced Solid Tumors	RECRUITING	Marengo Therapeutics, Inc. (Cambridge, MA, USA)		MultiCentric
NCT05572684	A Safety, Tolerability and Efficacy Study of NC410 Plus Pembrolizumab in Participants with Advanced Unresectable or Metastatic Solid Tumors	RECRUITING	NextCure, Inc. (Beltsville, MD, USA)	Merck Sharp & Dohme LLC	MultiCentric
NCT05559879	Cabozantinib and Dostarlimab in Recurrent Gynecologic Carcinosarcoma	RECRUITING	University of Alabama at Birmingham		O’Neal Comprehensive Cancer Center at UAB, Birmingham, AL, USA
NCT05520099	Observational Basket Trial to Collect Tissue to Train and Validate a Live Tumor Diagnostic Platform	RECRUITING	Elephas	Hoosier Cancer Research Network	MultiCentric
NCT05274451	A Study to Investigate LYL797 in Adults with Solid Tumors	RECRUITING	Lyell Immunopharma, Inc.		MultiCentric
NCT05269381	Personalized Neoantigen Peptide-Based Vaccine in Combination with Pembrolizumab for Treatment of Advanced Solid Tumors	RECRUITING	Mayo Clinic		Mayo Clinic in Florida, Jacksonville, FL, 32224-9980, USA
NCT05231122	Pembrolizumab Combined with Bevacizumab with or Without Agonist Anti-CD40 CDX-1140 for the Treatment of Patients With Recurrent Ovarian Cancer	RECRUITING	Roswell Park Cancer Institute	National Cancer Institute (NCI)|Merck Sharp & Dohme LLC|Celldex Therapeutics	Roswell Park Cancer Institute, Buffalo, New York, 14263, United States|M D Anderson Cancer Center, Houston, TX, USA
NCT05194735	Phase I/II Study of Autologous T Cells to Express T-Cell Receptors (TCRs) in Subjects with Solid Tumors	ACTIVE_NOT_RECRUITING	Alaunos Therapeutics		MD Anderson Cancer Center, Houston, TX, 77030, USA
NCT05112601	Testing Nivolumab with or Without Ipilimumab in Deficient Mismatch Repair System (dMMR) Recurrent Endometrial Carcinoma	RECRUITING	National Cancer Institute (NCI)	NRG Oncology	MultiCentric
NCT05092373	Phase I Study of Tumor Treating Fields (TTF) in Combination with Cabozantinib or With Pembrolizumab and Nab-Paclitaxel in Patients with Advanced Solid Tumors Involving the Abdomen or Thorax	RECRUITING	M.D. Anderson Cancer Center		M D Anderson Cancer Center, Houston, TX, 77030, USA
NCT05086692	A Beta-only IL-2 ImmunoTherapY Study	RECRUITING	Medicenna Therapeutics, Inc. (Toronto, ON, Canada)	Merck Sharp & Dohme LLC	MultiCentric
NCT04919629	APL-2 and Pembrolizumab Versus APL-2, Pembrolizumab and Bevacizumab Versus Bevacizumab Alone for the Treatment of Recurrent Ovarian, Fallopian Tube, or Primary Peritoneal Cancer and Malignant Effusion	RECRUITING	Roswell Park Cancer Institute	National Cancer Institute (NCI)	Roswell Park Cancer Institute, Buffalo, NY, 14263, USA
NCT04670445	Improving Patient and Caregiver Understanding of Risks and Benefits of Immunotherapy for Advanced Cancer	ACTIVE_NOT_RECRUITING	Massachusetts General Hospital	Conquer Cancer Foundation	Massachusetts General Hospital, Boston, MA, 02115, USA
NCT04660929	CAR-macrophages for the Treatment of HER2 Overexpressing Solid Tumors	ACTIVE_NOT_RECRUITING	Carisma Therapeutics Inc. (Philadelphia, PA, USA)		MultiCentric
NCT04486352	A Study of Targeted Agents for Patients with Recurrent or Persistent Endometrial Cancer	RECRUITING	Alliance Foundation Trials, LLC.	Genentech, Inc.|Foundation Medicine|Pfizer|Eli Lilly and Company	MultiCentric
NCT04463771	Safety and Efficacy of Retifanlimab (INCMGA00012) Alone or in Combination with Other Therapies in Participants With Advanced or Metastatic Endometrial Cancer Who Have Progressed on or After Platinum-based Chemotherapy	ACTIVE_NOT_RECRUITING	Incyte Corporation	GOG Foundation|European Network of Gynaecological Oncological Trial Groups (ENGOT)	MultiCentric
NCT04319757	ACE1702 in Subjects with Advanced or Metastatic HER2-expressing Solid Tumors	RECRUITING	Acepodia Biotech, Inc. (Alameda, CA, USA)		MultiCentric
NCT04278144	A First-in-human Study Using BDC-1001 as a Single Agent and in Combination with Nivolumab in Advanced HER2-Expressing Solid Tumors	ACTIVE_NOT_RECRUITING	Bolt Biotherapeutics, Inc. (Redwood City, CA, USA)	Bristol-Myers Squibb	MultiCentric
NCT04272034	Safety, Tolerability, Pharmacokinetics, and Pharmacodynamics of INCB099318 in Participants with Advanced Solid Tumors	ACTIVE_NOT_RECRUITING	Incyte Corporation		MultiCentric
NCT04214067	Testing the Addition of the Immunotherapy Drug, Pembrolizumab, to the Usual Radiation Treatment for Newly Diagnosed early-stage High Intermediate Risk Endometrial Cancer	ACTIVE_NOT_RECRUITING	National Cancer Institute (NCI)	NRG Oncology	MultiCentric
NCT04034927	Testing the Addition of an Immunotherapy Drug, Tremelimumab, to the PARP Inhibition Drug, Olaparib, for Recurrent Ovarian, Fallopian Tube or Peritoneal Cancer	ACTIVE_NOT_RECRUITING	National Cancer Institute (NCI)	NRG Oncology	MultiCentric
NCT03932409	Frontline Immunotherapy Combined with Radiation and Chemotherapy in High Risk Endometrial Cancer	RECRUITING	University of Oklahoma	Merck Sharp & Dohme LLC	LSU Health New Orleans, New Orleans, Louisiana, 70112, United States|Stephenson Cancer Center, Oklahoma City, OK, 73104, USA
NCT03914612	Testing the Addition of the Immunotherapy Drug Pembrolizumab to the Usual Chemotherapy Treatment (Paclitaxel and Carboplatin) in Stage III-IV or Recurrent Endometrial Cancer	ACTIVE_NOT_RECRUITING	National Cancer Institute (NCI)	Canadian Cancer Trials Group|NRG Oncology	MultiCentric
NCT03860272	Fc-Engineered Anti-CTLA-4 Monoclonal Antibody in Advanced Cancer	RECRUITING	Agenus Inc. (Lexington, MA, USA)		MultiCentric
NCT03835819	A Phase 2 Study of Mirvetuximab Soravtansine (IMGN853) and Pembrolizumab in Endometrial Cancer (EC)	ACTIVE_NOT_RECRUITING	Dana-Farber Cancer Institute	ImmunoGen, Inc.|Merck Sharp & Dohme LLC	Dana Farber Cancer Institute, Boston, Massachusetts, 02215, United States|University of Massachusetts, Worcester, Massachusetts, 01605, United States|Northwell Cancer Institute, Lake Success, NY, 11042, USA
NCT03508570	Nivolumab with or Without Ipilimumab in treating patients with Recurrent or high grade gynecologic cancer with Metastatic Peritoneal Carcinomatosis	ACTIVE_NOT_RECRUITING	M.D. Anderson Cancer Center	National Cancer Institute (NCI)	M D Anderson Cancer Center, Houston, TX, 77030, USA
NCT03452774	SYNERGY-AI: Artificial Intelligence Based Precision Oncology Clinical Trial Matching and Registry	RECRUITING	Massive Bio, Inc. (New York, NY, USA)		Massive Bio, Inc, New York, NY, 10006, USA
NCT03367741	Cabozantinib S-malate and Nivolumab in Treating Patients with Advanced, Recurrent, or Metastatic Endometrial Cancer	ACTIVE_NOT_RECRUITING	National Cancer Institute (NCI)		MultiCentric
NCT02839707	Pegylated Liposomal Doxorubicin Hydrochloride with Atezolizumab and/or Bevacizumab in Treating Patients with Recurrent Ovarian, Fallopian Tube, or Primary Peritoneal Cancer	ACTIVE_NOT_RECRUITING	National Cancer Institute (NCI)	NRG Oncology	MultiCentric
NCT02834013	Nivolumab and Ipilimumab in Treating Patients with Rare Tumors	ACTIVE_NOT_RECRUITING	National Cancer Institute (NCI)		MultiCentric
NCT02715284	Study of TSR-042, an Anti-programmed Cell Death-1 Receptor (PD-1) Monoclonal Antibody, in Participants with Advanced Solid Tumors	RECRUITING	Tesaro, Inc. (Waltham, MA, USA)		MultiCentric
NCT01174121	Immunotherapy Using Tumor Infiltrating Lymphocytes for Patients with Metastatic Cancer	RECRUITING	National Cancer Institute (NCI)		National Institutes of Health Clinical Center, Bethesda, MD, 20892, USA
NCT00565851	Carboplatin, Paclitaxel and Gemcitabine Hydrochloride with or Without Bevacizumab After Surgery in Treating Patients with Recurrent Ovarian, Epithelial, Primary Peritoneal, or Fallopian Tube Cancer	ACTIVE_NOT_RECRUITING	National Cancer Institute (NCI)	NRG Oncology	MultiCentric
